# 1,3-Bis(2,6-diisopropyl­phen­yl)imidazolidin-2-yl­idene

**DOI:** 10.1107/S1600536810029922

**Published:** 2010-08-04

**Authors:** Nick A. Giffin, Arthur D. Hendsbee, Jason D. Masuda

**Affiliations:** a The Maritime Centre for Green Chemistry (MCGC), Department of Chemistry, Saint Mary’s University, 923 Robie Street, Halifax, NS B3H 3C3, Canada

## Abstract

The title compound, C_27_H_38_N_2_, is the first reported free imidazolidin-2-yl­idene carbene with 2,6-diisopropyl­phenyl groups in the 1,3-positions. The five-membered ring adopts a twisted conformation and the dihedral angle between the aromatic rings is 48.81 (6)°. Both isopropyl groups attached to one of the benzene rings are disordered over two sets of sites in 0.74 (2):0.26 (2) and 0.599 (8):0.401 (8) ratios.

## Related literature

There are few examples in the literature of crystallograph­ic­ally characterized free ylidenes with *ortho*-alkyl substituted phenyl groups in the 1,3-positions: for related structures see: Arduengo *et al.* (1991 ▶, 1992 ▶, 1995 ▶, 1999 ▶). For background to free carbenes, see: Igau *et al.* (1989 ▶) and for Arduengo-type carbenes, see: Pauling (1980 ▶).
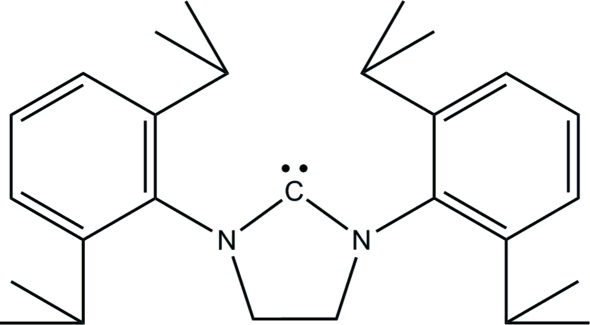


## Experimental

### 

#### Crystal data

C_27_H_38_N_2_
*M* *_r_* = 390.59
Monoclinic, 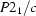

*a* = 20.835 (7) Å
*b* = 5.922 (2) Å
*c* = 19.694 (7) Å
β = 93.090 (4)°
*V* = 2426.2 (14) Å^3^
*Z* = 4
Mo *K*α radiation
μ = 0.06 mm^−1^
*T* = 150 K
0.50 × 0.34 × 0.12 mm

#### Data collection

Bruker APEXII CCD diffractometer
Absorption correction: multi-scan (*SADABS*; Bruker, 2008 ▶) *T*_min_ = 0.716, *T*_max_ = 0.746
22607 measured reflections
4259 independent reflections
3326 reflections with *I* > 2σ(*I*)
*R*_int_ = 0.033

#### Refinement

*R*[*F* ^2^ > 2σ(*F* ^2^)] = 0.037
*wR*(*F* ^2^) = 0.097
*S* = 1.01
4259 reflections
319 parameters
168 restraints
H-atom parameters constrained
Δρ_max_ = 0.18 e Å^−3^
Δρ_min_ = −0.14 e Å^−3^

### 

Data collection: *APEX2* (Bruker, 2008 ▶); cell refinement: *SAINT* (Bruker, 2008 ▶); data reduction: *SAINT*; program(s) used to solve structure: *SHELXS97* (Sheldrick, 2008 ▶); program(s) used to refine structure: *SHELXL97* (Sheldrick, 2008 ▶); molecular graphics: *ORTEP-3* (Farrugia, 1997 ▶); software used to prepare material for publication: *SHELXTL* (Sheldrick, 2008 ▶).

## Supplementary Material

Crystal structure: contains datablocks I, global. DOI: 10.1107/S1600536810029922/hb5563sup1.cif

Structure factors: contains datablocks I. DOI: 10.1107/S1600536810029922/hb5563Isup2.hkl

Additional supplementary materials:  crystallographic information; 3D view; checkCIF report

## Figures and Tables

**Fig. 1.**
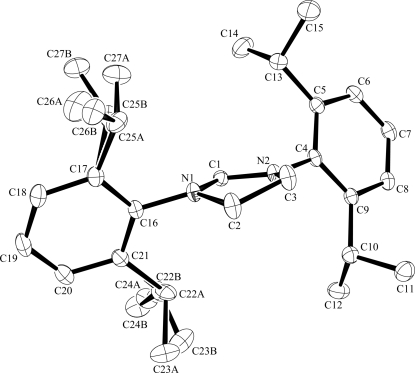
The molecular structure of (I) with 30% probability displacement ellipsoids for non-H atoms. Perspective is down the 010 axis and H atoms are removed for clarity.

## supplementary crystallographic information

### Comment

Free carbenes have received substantial attention in the literature since their introduction by Igau *et al.* (1989). Arduengo-type carbenes, described by Linus Pauling as push-push, mesomeric pull-pull (Pauling, 1980) are electronically stabilized through donating amino-substituents and sterically protected by alkyl substituted phenyl groups in the 1,3-positions. Beginning in 1991, free diamino carbenes such as the 1,3-*bis*(1-adamantyl)imidazol-2-ylidiene (Arduengo *et al.*, 1991) have garnered substantial notoriety across chemical disciplines.

As a result of the increased steric bulk associated with the flanking 2,6-diisopropylphenyl substiuents the title free carbene exibits a N1—C1—N2 bond angle of 104.98 (11)°. This angle represents substantial relaxation when compared to the IPr carbene, 1,3-*bis*(2,6-diisopropylphenyl)imidazol-2-ylidiene, 101.4 (2)° and the IMes carbene 1,3-*bis* (2,4,6-trimethylphenyl)imidazolidin-2-ylidiene, 101.4 (3)° (Arduengo *et al.*, 1999). However, the N1—C1—N2 angle in the title molecule is similar to that of the saturated analogue of IMes, 1,3-*bis*(2,4,6-trimethylphenyl)imidazolidin-2-ylidiene, 104.7 (3)° (Arduengo *et al.*, 1995). It should be noted that the unit-cell parameters are nearly identical to those reported for the analagous IPr carbene (Arduengo *et al.*, 1999). This is not surprising as the addition of two Hydrogen atoms to the C=C bond in the backbone of the molecule will casue little change in the overall molecular volume and shape of the parent molecule relative to that of the imidazol-2-ylidiene.

### Experimental

1,3-*bis*(2,6-diisopropylphenyl)imidazolidin-2-ylidene was prepared through the addition of 0.466 g of potassium *bis*-hexamethyl disilazide to a solution of 1.00 g (0.234 mmol) of 1,3-*bis*(2,6-diisopropylphenyl) imidazolidinium chloride (0.234 mmol) in diethylether. Volatiles were removed under reduced pressure and the remaining solid was dissolved in pentane, filtered through diatomaceous earth and cooled to 243 K yielding colorless blocks of (I). The proton NMR matched that in the literature of the title ylidene (Arduengo *et al.*, 1999).

### Refinement

The H atoms were placed in geometrically idealized positions with C—H distances of 0.95Å (aromatic),0.98Å (idealized tertiary), 0.99Å (Idealized secondary) and 0.98Å (Idealized methyl). H atoms were constrained to ride on the parent C atom with *U*_iso_(H) = 1.2Ueq(C) for aromatic, *U*_iso_(H) = 1.5Ueq(C) for the idealized methyl protons, *U*_iso_(H) = 1.2Ueq(C) for the idealized tertiary protons and *U*_iso_(H) = 1.2Ueq(C) for the idealized secondary protons. A short contact distance of 1.89 Angstroms is observed between H31B and H2B, where H31B lies in the disordered part of the model. Tests for twinning and missed symmetry were preformed and no twinning laws or change of spacegroup were suggested. The short contact is believed to arise from the disorder present in the crystal. In order to obtain satisfactory thermal parametersthe use of SIMU and DELU restraints were applied to carbon atoms C21 to C27.

### Crystal data

**Table:**

C_27_H_38_N_2_	*F*(000) = 856
*M**_r_* = 390.59	*D*_x_ = 1.069 Mg m^−^^3^
Monoclinic, *P*2_1_/*c*	Mo *K*α radiation, λ = 0.71073 Å
Hall symbol: -P 2ybc	Cell parameters from 9396 reflections
*a* = 20.835 (7) Å	θ = 2.2–28.4°
*b* = 5.922 (2) Å	µ = 0.06 mm^−^^1^
*c* = 19.694 (7) Å	*T* = 150 K
β = 93.090 (4)°	Block, colourless
*V* = 2426.2 (14) Å^3^	0.50 × 0.34 × 0.12 mm
*Z* = 4	

### Data collection

**Table:**

Bruker APEXII CCD diffractometer	4259 independent reflections
Radiation source: fine-focus sealed tube	3326 reflections with *I* > 2σ(*I*)
graphite	*R*_int_ = 0.033
φ and ω scans	θ_max_ = 25.0°, θ_min_ = 2.1°
Absorption correction: multi-scan (*SADABS*; Bruker, 2008)	*h* = −24→24
*T*_min_ = 0.716, *T*_max_ = 0.746	*k* = −7→7
22607 measured reflections	*l* = −23→23

### Refinement

**Table:**

Refinement on *F*^2^	Secondary atom site location: difference Fourier map
Least-squares matrix: full	Hydrogen site location: inferred from neighbouring sites
*R*[*F*^2^ > 2σ(*F*^2^)] = 0.037	H-atom parameters constrained
*wR*(*F*^2^) = 0.097	*w* = 1/[σ^2^(*F*_o_^2^) + (0.0419*P*)^2^ + 0.6416*P*] where *P* = (*F*_o_^2^ + 2*F*_c_^2^)/3
*S* = 1.01	(Δ/σ)_max_ = 0.002
4259 reflections	Δρ_max_ = 0.18 e Å^−^^3^
319 parameters	Δρ_min_ = −0.14 e Å^−^^3^
168 restraints	Extinction correction: *SHELXL97* (Sheldrick, 2008), Fc^*^=kFc[1+0.001xFc^2^λ^3^/sin(2θ)]^-1/4^
Primary atom site location: structure-invariant direct methods	Extinction coefficient: 0.0031 (6)

### Special details

**Table:**

Geometry. All e.s.d.'s (except the e.s.d. in the dihedral angle between two l.s. planes) are estimated using the full covariance matrix. The cell e.s.d.'s are taken into account individually in the estimation of e.s.d.'s in distances, angles and torsion angles; correlations between e.s.d.'s in cell parameters are only used when they are defined by crystal symmetry. An approximate (isotropic) treatment of cell e.s.d.'s is used for estimating e.s.d.'s involving l.s. planes.
Refinement. Refinement of *F*^2^ against ALL reflections. The weighted *R*-factor *wR* and goodness of fit *S* are based on *F*^2^, conventional *R*-factors *R* are based on *F*, with *F* set to zero for negative *F*^2^. The threshold expression of *F*^2^ > σ(*F*^2^) is used only for calculating *R*-factors(gt) *etc*. and is not relevant to the choice of reflections for refinement. *R*-factors based on *F*^2^ are statistically about twice as large as those based on *F*, and *R*- factors based on ALL data will be even larger.

### Fractional atomic coordinates and isotropic or equivalent isotropic displacement parameters (Å^2^)

**Table:**

	*x*	*y*	*z*	*U*_iso_*/*U*_eq_	Occ. (<1)
N1	0.21083 (5)	0.66151 (18)	0.15446 (5)	0.0270 (3)	
N2	0.30506 (5)	0.79278 (17)	0.17711 (5)	0.0263 (3)	
C1	0.24262 (6)	0.8528 (2)	0.17156 (6)	0.0252 (3)	
C2	0.25298 (6)	0.4697 (2)	0.13858 (8)	0.0379 (4)	
H2A	0.2557	0.4496	0.0889	0.046*	
H2B	0.2382	0.3272	0.1589	0.046*	
C3	0.31647 (6)	0.5471 (2)	0.17175 (8)	0.0369 (4)	
H3A	0.3248	0.4771	0.2170	0.044*	
H3B	0.3528	0.5136	0.1429	0.044*	
C4	0.35630 (6)	0.9431 (2)	0.19756 (6)	0.0251 (3)	
C5	0.36374 (6)	1.0164 (2)	0.26515 (7)	0.0286 (3)	
C6	0.41414 (6)	1.1635 (2)	0.28223 (7)	0.0327 (3)	
H6A	0.4197	1.2172	0.3276	0.039*	
C7	0.45624 (6)	1.2329 (2)	0.23476 (7)	0.0330 (3)	
H7A	0.4904	1.3331	0.2475	0.040*	
C8	0.44864 (6)	1.1564 (2)	0.16868 (7)	0.0301 (3)	
H8A	0.4779	1.2043	0.1363	0.036*	
C9	0.39891 (6)	1.0107 (2)	0.14862 (6)	0.0268 (3)	
C10	0.39200 (6)	0.9307 (2)	0.07521 (7)	0.0324 (3)	
H10A	0.3536	0.8297	0.0707	0.039*	
C11	0.45050 (7)	0.7930 (3)	0.05695 (8)	0.0427 (4)	
H11A	0.4566	0.6670	0.0889	0.064*	
H11B	0.4888	0.8896	0.0596	0.064*	
H11C	0.4437	0.7340	0.0106	0.064*	
C12	0.38055 (8)	1.1287 (3)	0.02624 (7)	0.0461 (4)	
H12A	0.3424	1.2128	0.0387	0.069*	
H12B	0.3739	1.0714	−0.0203	0.069*	
H12C	0.4180	1.2290	0.0290	0.069*	
C13	0.31902 (7)	0.9415 (3)	0.31916 (7)	0.0366 (3)	
H13A	0.2890	0.8257	0.2984	0.044*	
C14	0.27892 (8)	1.1385 (3)	0.34266 (9)	0.0580 (5)	
H14A	0.2547	1.2044	0.3035	0.087*	
H14B	0.3073	1.2533	0.3639	0.087*	
H14C	0.2489	1.0847	0.3757	0.087*	
C15	0.35625 (8)	0.8326 (3)	0.37951 (8)	0.0608 (5)	
H15A	0.3810	0.7042	0.3636	0.091*	
H15B	0.3260	0.7799	0.4125	0.091*	
H15C	0.3856	0.9438	0.4011	0.091*	
C16	0.14366 (6)	0.6519 (2)	0.13366 (6)	0.0268 (3)	
C17	0.10301 (6)	0.5242 (2)	0.17292 (7)	0.0320 (3)	
C18	0.03883 (6)	0.5047 (3)	0.15029 (8)	0.0390 (4)	
H18A	0.0104	0.4177	0.1759	0.047*	
C19	0.01594 (7)	0.6089 (3)	0.09170 (8)	0.0431 (4)	
H19A	−0.0280	0.5931	0.0770	0.052*	
C20	0.05626 (7)	0.7360 (3)	0.05408 (7)	0.0408 (4)	
H20A	0.0396	0.8082	0.0138	0.049*	
C21	0.12121 (6)	0.7610 (2)	0.07385 (7)	0.0317 (3)	
C22B	0.1629 (5)	0.9020 (16)	0.0300 (6)	0.0390 (10)	0.74 (2)
H22A	0.2022	0.9410	0.0591	0.047*	0.74 (2)
C23B	0.1861 (4)	0.7654 (14)	−0.0289 (4)	0.0624 (15)	0.74 (2)
H23A	0.2075	0.6282	−0.0116	0.094*	0.74 (2)
H23B	0.2164	0.8558	−0.0539	0.094*	0.74 (2)
H23C	0.1493	0.7240	−0.0595	0.094*	0.74 (2)
C24B	0.1334 (4)	1.1277 (10)	0.0058 (4)	0.0546 (15)	0.74 (2)
H24A	0.1183	1.2108	0.0449	0.082*	0.74 (2)
H24B	0.0972	1.0989	−0.0269	0.082*	0.74 (2)
H24C	0.1660	1.2175	−0.0161	0.082*	0.74 (2)
C22A	0.1707 (15)	0.880 (4)	0.0293 (14)	0.037 (2)	0.26 (2)
H22B	0.2152	0.8825	0.0512	0.045*	0.26 (2)
C23A	0.1669 (12)	0.755 (4)	−0.0425 (9)	0.056 (3)	0.26 (2)
H23D	0.1970	0.8264	−0.0726	0.083*	0.26 (2)
H23E	0.1231	0.7667	−0.0630	0.083*	0.26 (2)
H23F	0.1783	0.5958	−0.0361	0.083*	0.26 (2)
C24A	0.1448 (11)	1.106 (3)	0.0164 (12)	0.044 (3)	0.26 (2)
H24D	0.1483	1.1944	0.0585	0.066*	0.26 (2)
H24E	0.0995	1.0942	0.0006	0.066*	0.26 (2)
H24F	0.1690	1.1802	−0.0185	0.066*	0.26 (2)
C25B	0.1227 (4)	0.4253 (16)	0.2422 (5)	0.0339 (9)	0.599 (8)
H25A	0.1698	0.4534	0.2505	0.041*	0.599 (8)
C26B	0.1126 (4)	0.1722 (14)	0.2434 (5)	0.0444 (11)	0.599 (8)
H26A	0.1338	0.1030	0.2053	0.067*	0.599 (8)
H26B	0.0665	0.1392	0.2392	0.067*	0.599 (8)
H26C	0.1310	0.1106	0.2864	0.067*	0.599 (8)
C27B	0.0885 (3)	0.5341 (5)	0.30107 (14)	0.0470 (10)	0.599 (8)
H27A	0.0932	0.6986	0.2989	0.070*	0.599 (8)
H27B	0.1077	0.4787	0.3445	0.070*	0.599 (8)
H27C	0.0428	0.4945	0.2974	0.070*	0.599 (8)
C25A	0.1323 (7)	0.398 (2)	0.2352 (7)	0.0383 (16)	0.401 (8)
H25B	0.1779	0.3604	0.2266	0.046*	0.401 (8)
C26A	0.0966 (7)	0.178 (2)	0.2496 (10)	0.065 (3)	0.401 (8)
H26D	0.1186	0.0995	0.2882	0.097*	0.401 (8)
H26E	0.0961	0.0802	0.2094	0.097*	0.401 (8)
H26F	0.0523	0.2121	0.2606	0.097*	0.401 (8)
C27A	0.1321 (4)	0.5605 (9)	0.2935 (2)	0.0548 (18)	0.401 (8)
H27D	0.1528	0.4903	0.3340	0.082*	0.401 (8)
H27E	0.0876	0.6000	0.3024	0.082*	0.401 (8)
H27F	0.1556	0.6974	0.2821	0.082*	0.401 (8)

### Atomic displacement parameters (Å^2^)

**Table:**

	*U*^11^	*U*^22^	*U*^33^	*U*^12^	*U*^13^	*U*^23^
N1	0.0214 (5)	0.0227 (6)	0.0366 (6)	0.0006 (5)	−0.0017 (4)	0.0014 (5)
N2	0.0228 (5)	0.0192 (6)	0.0366 (6)	0.0009 (4)	−0.0017 (4)	0.0016 (5)
C1	0.0237 (6)	0.0264 (7)	0.0252 (6)	0.0000 (5)	0.0002 (5)	0.0032 (5)
C2	0.0277 (7)	0.0221 (7)	0.0631 (10)	0.0019 (6)	−0.0051 (7)	−0.0008 (7)
C3	0.0273 (7)	0.0222 (7)	0.0604 (10)	0.0013 (6)	−0.0051 (6)	0.0017 (7)
C4	0.0214 (6)	0.0200 (6)	0.0333 (7)	0.0017 (5)	−0.0039 (5)	0.0019 (5)
C5	0.0268 (7)	0.0271 (7)	0.0314 (7)	0.0055 (6)	−0.0025 (5)	0.0023 (6)
C6	0.0329 (7)	0.0307 (8)	0.0335 (7)	0.0045 (6)	−0.0076 (6)	−0.0036 (6)
C7	0.0271 (7)	0.0255 (7)	0.0454 (8)	−0.0016 (6)	−0.0087 (6)	−0.0008 (6)
C8	0.0236 (7)	0.0277 (7)	0.0390 (8)	−0.0006 (6)	0.0004 (5)	0.0050 (6)
C9	0.0234 (6)	0.0242 (7)	0.0323 (7)	0.0035 (5)	−0.0020 (5)	0.0023 (6)
C10	0.0300 (7)	0.0345 (8)	0.0328 (7)	−0.0010 (6)	0.0016 (6)	−0.0018 (6)
C11	0.0435 (9)	0.0424 (9)	0.0427 (9)	0.0055 (7)	0.0069 (7)	−0.0045 (7)
C12	0.0572 (10)	0.0485 (10)	0.0323 (8)	0.0104 (8)	−0.0014 (7)	0.0008 (7)
C13	0.0358 (8)	0.0432 (9)	0.0308 (7)	0.0016 (7)	0.0011 (6)	0.0052 (6)
C14	0.0544 (10)	0.0698 (12)	0.0518 (10)	0.0190 (9)	0.0202 (8)	0.0124 (9)
C15	0.0571 (11)	0.0747 (13)	0.0510 (10)	0.0123 (10)	0.0059 (8)	0.0300 (10)
C16	0.0222 (6)	0.0249 (7)	0.0329 (7)	0.0004 (5)	−0.0017 (5)	−0.0027 (6)
C17	0.0276 (7)	0.0295 (7)	0.0389 (7)	−0.0019 (6)	0.0014 (6)	−0.0008 (6)
C18	0.0272 (7)	0.0397 (9)	0.0505 (9)	−0.0065 (6)	0.0053 (6)	−0.0013 (7)
C19	0.0238 (7)	0.0504 (10)	0.0542 (10)	−0.0020 (7)	−0.0073 (7)	−0.0055 (8)
C20	0.0354 (8)	0.0459 (9)	0.0397 (8)	0.0025 (7)	−0.0100 (7)	0.0000 (7)
C21	0.0302 (7)	0.0328 (7)	0.0317 (7)	0.0006 (6)	−0.0023 (5)	−0.0022 (6)
C22B	0.029 (2)	0.048 (2)	0.0391 (18)	0.0014 (14)	−0.0040 (14)	0.0110 (14)
C23B	0.071 (3)	0.055 (2)	0.064 (3)	0.016 (2)	0.032 (3)	0.016 (2)
C24B	0.075 (4)	0.0357 (17)	0.054 (3)	0.0053 (15)	0.009 (2)	0.0093 (14)
C22A	0.047 (6)	0.037 (5)	0.027 (4)	−0.004 (4)	−0.005 (5)	0.011 (3)
C23A	0.088 (8)	0.045 (5)	0.035 (4)	0.006 (6)	0.014 (5)	0.001 (4)
C24A	0.053 (6)	0.034 (4)	0.044 (6)	−0.003 (4)	−0.003 (5)	0.002 (3)
C25B	0.024 (2)	0.0335 (19)	0.0445 (17)	0.0001 (19)	0.0031 (16)	0.0070 (17)
C26B	0.044 (3)	0.0332 (15)	0.057 (3)	0.0022 (19)	0.009 (2)	0.0067 (13)
C27B	0.063 (3)	0.0396 (15)	0.0390 (12)	0.0043 (16)	0.0089 (15)	0.0040 (11)
C25A	0.040 (5)	0.035 (4)	0.041 (3)	0.003 (3)	0.012 (2)	0.0096 (16)
C26A	0.073 (7)	0.044 (3)	0.079 (5)	−0.010 (4)	0.018 (5)	0.020 (3)
C27A	0.075 (5)	0.052 (2)	0.0371 (18)	0.009 (3)	−0.001 (2)	0.005 (2)

### Geometric parameters (Å, °)

**Table:**

N1—C1	1.3458 (16)	C18—C19	1.371 (2)
N1—C16	1.4380 (16)	C18—H18A	0.9500
N1—C2	1.4793 (17)	C19—C20	1.374 (2)
N2—C1	1.3474 (16)	C19—H19A	0.9500
N2—C4	1.4309 (16)	C20—C21	1.3959 (19)
N2—C3	1.4790 (17)	C20—H20A	0.9500
C2—C3	1.5144 (19)	C21—C22B	1.509 (9)
C2—H2A	0.9900	C21—C22A	1.56 (3)
C2—H2B	0.9900	C22B—C23B	1.515 (9)
C3—H3A	0.9900	C22B—C24B	1.537 (6)
C3—H3B	0.9900	C22B—H22A	1.0000
C4—C5	1.4007 (18)	C23B—H23A	0.9800
C4—C9	1.4034 (18)	C23B—H23B	0.9800
C5—C6	1.3915 (19)	C23B—H23C	0.9800
C5—C13	1.5176 (19)	C24B—H24A	0.9800
C6—C7	1.379 (2)	C24B—H24B	0.9800
C6—H6A	0.9500	C24B—H24C	0.9800
C7—C8	1.3791 (19)	C22A—C24A	1.458 (17)
C7—H7A	0.9500	C22A—C23A	1.59 (3)
C8—C9	1.3894 (18)	C22A—H22B	1.0000
C8—H8A	0.9500	C23A—H23D	0.9800
C9—C10	1.5205 (19)	C23A—H23E	0.9800
C10—C11	1.526 (2)	C23A—H23F	0.9800
C10—C12	1.529 (2)	C24A—H24D	0.9800
C10—H10A	1.0000	C24A—H24E	0.9800
C11—H11A	0.9800	C24A—H24F	0.9800
C11—H11B	0.9800	C25B—C26B	1.514 (11)
C11—H11C	0.9800	C25B—C27B	1.535 (6)
C12—H12A	0.9800	C25B—H25A	1.0000
C12—H12B	0.9800	C26B—H26A	0.9800
C12—H12C	0.9800	C26B—H26B	0.9800
C13—C14	1.521 (2)	C26B—H26C	0.9800
C13—C15	1.527 (2)	C27B—H27A	0.9800
C13—H13A	1.0000	C27B—H27B	0.9800
C14—H14A	0.9800	C27B—H27C	0.9800
C14—H14B	0.9800	C25A—C27A	1.497 (10)
C14—H14C	0.9800	C25A—C26A	1.537 (18)
C15—H15A	0.9800	C25A—H25B	1.0000
C15—H15B	0.9800	C26A—H26D	0.9800
C15—H15C	0.9800	C26A—H26E	0.9800
C16—C17	1.3992 (19)	C26A—H26F	0.9800
C16—C21	1.4015 (18)	C27A—H27D	0.9800
C17—C18	1.3913 (19)	C27A—H27E	0.9800
C17—C25B	1.521 (11)	C27A—H27F	0.9800
C17—C25A	1.534 (18)		
			
C1—N1—C16	124.27 (10)	C18—C19—C20	120.34 (13)
C1—N1—C2	114.15 (10)	C18—C19—H19A	119.8
C16—N1—C2	119.28 (10)	C20—C19—H19A	119.8
C1—N2—C4	124.14 (11)	C19—C20—C21	121.30 (14)
C1—N2—C3	114.32 (10)	C19—C20—H20A	119.3
C4—N2—C3	120.77 (10)	C21—C20—H20A	119.3
N1—C1—N2	104.98 (11)	C20—C21—C16	117.39 (13)
N1—C2—C3	101.04 (11)	C20—C21—C22B	118.6 (4)
N1—C2—H2A	111.6	C16—C21—C22B	124.0 (4)
C3—C2—H2A	111.6	C20—C21—C22A	123.5 (12)
N1—C2—H2B	111.6	C16—C21—C22A	118.7 (11)
C3—C2—H2B	111.6	C21—C22B—C23B	111.1 (7)
H2A—C2—H2B	109.4	C21—C22B—C24B	115.2 (6)
N2—C3—C2	100.85 (10)	C23B—C22B—C24B	111.4 (8)
N2—C3—H3A	111.6	C21—C22B—H22A	106.2
C2—C3—H3A	111.6	C23B—C22B—H22A	106.2
N2—C3—H3B	111.6	C24B—C22B—H22A	106.2
C2—C3—H3B	111.6	C22B—C23B—H23A	109.5
H3A—C3—H3B	109.4	C22B—C23B—H23B	109.5
C5—C4—C9	121.50 (12)	H23A—C23B—H23B	109.5
C5—C4—N2	120.20 (11)	C22B—C23B—H23C	109.5
C9—C4—N2	118.29 (11)	H23A—C23B—H23C	109.5
C6—C5—C4	117.85 (12)	H23B—C23B—H23C	109.5
C6—C5—C13	119.82 (12)	C22B—C24B—H24A	109.5
C4—C5—C13	122.33 (12)	C22B—C24B—H24B	109.5
C7—C6—C5	121.48 (13)	H24A—C24B—H24B	109.5
C7—C6—H6A	119.3	C22B—C24B—H24C	109.5
C5—C6—H6A	119.3	H24A—C24B—H24C	109.5
C6—C7—C8	119.80 (12)	H24B—C24B—H24C	109.5
C6—C7—H7A	120.1	C24A—C22A—C21	105.2 (18)
C8—C7—H7A	120.1	C24A—C22A—C23A	106 (2)
C7—C8—C9	121.22 (13)	C21—C22A—C23A	107 (2)
C7—C8—H8A	119.4	C24A—C22A—H22B	112.9
C9—C8—H8A	119.4	C21—C22A—H22B	112.9
C8—C9—C4	118.13 (12)	C23A—C22A—H22B	112.9
C8—C9—C10	119.64 (12)	C22A—C23A—H23D	109.5
C4—C9—C10	122.22 (11)	C22A—C23A—H23E	109.5
C9—C10—C11	110.78 (11)	H23D—C23A—H23E	109.5
C9—C10—C12	111.37 (12)	C22A—C23A—H23F	109.5
C11—C10—C12	111.15 (12)	H23D—C23A—H23F	109.5
C9—C10—H10A	107.8	H23E—C23A—H23F	109.5
C11—C10—H10A	107.8	C22A—C24A—H24D	109.5
C12—C10—H10A	107.8	C22A—C24A—H24E	109.5
C10—C11—H11A	109.5	H24D—C24A—H24E	109.5
C10—C11—H11B	109.5	C22A—C24A—H24F	109.5
H11A—C11—H11B	109.5	H24D—C24A—H24F	109.5
C10—C11—H11C	109.5	H24E—C24A—H24F	109.5
H11A—C11—H11C	109.5	C26B—C25B—C17	111.3 (7)
H11B—C11—H11C	109.5	C26B—C25B—C27B	109.5 (7)
C10—C12—H12A	109.5	C17—C25B—C27B	113.7 (6)
C10—C12—H12B	109.5	C26B—C25B—H25A	107.4
H12A—C12—H12B	109.5	C17—C25B—H25A	107.4
C10—C12—H12C	109.5	C27B—C25B—H25A	107.4
H12A—C12—H12C	109.5	C25B—C26B—H26A	109.5
H12B—C12—H12C	109.5	C25B—C26B—H26B	109.5
C5—C13—C14	110.94 (12)	H26A—C26B—H26B	109.5
C5—C13—C15	111.32 (12)	C25B—C26B—H26C	109.5
C14—C13—C15	110.57 (13)	H26A—C26B—H26C	109.5
C5—C13—H13A	108.0	H26B—C26B—H26C	109.5
C14—C13—H13A	108.0	C25B—C27B—H27A	109.5
C15—C13—H13A	108.0	C25B—C27B—H27B	109.5
C13—C14—H14A	109.5	H27A—C27B—H27B	109.5
C13—C14—H14B	109.5	C25B—C27B—H27C	109.5
H14A—C14—H14B	109.5	H27A—C27B—H27C	109.5
C13—C14—H14C	109.5	H27B—C27B—H27C	109.5
H14A—C14—H14C	109.5	C27A—C25A—C17	106.4 (9)
H14B—C14—H14C	109.5	C27A—C25A—C26A	112.5 (11)
C13—C15—H15A	109.5	C17—C25A—C26A	112.7 (12)
C13—C15—H15B	109.5	C27A—C25A—H25B	108.4
H15A—C15—H15B	109.5	C17—C25A—H25B	108.4
C13—C15—H15C	109.5	C26A—C25A—H25B	108.4
H15A—C15—H15C	109.5	C25A—C26A—H26D	109.5
H15B—C15—H15C	109.5	C25A—C26A—H26E	109.5
C17—C16—C21	121.95 (12)	H26D—C26A—H26E	109.5
C17—C16—N1	118.22 (11)	C25A—C26A—H26F	109.5
C21—C16—N1	119.78 (11)	H26D—C26A—H26F	109.5
C18—C17—C16	117.89 (13)	H26E—C26A—H26F	109.5
C18—C17—C25B	117.6 (4)	C25A—C27A—H27D	109.5
C16—C17—C25B	124.2 (4)	C25A—C27A—H27E	109.5
C18—C17—C25A	123.3 (6)	H27D—C27A—H27E	109.5
C16—C17—C25A	118.6 (6)	C25A—C27A—H27F	109.5
C19—C18—C17	121.12 (13)	H27D—C27A—H27F	109.5
C19—C18—H18A	119.4	H27E—C27A—H27F	109.5
C17—C18—H18A	119.4		
